# Diabetes Complications and Comorbidities as Risk Factors for MACE in People With Type 2 Diabetes and Their Development Over Time: A Danish Registry‐Based Case–Control Study

**DOI:** 10.1111/1753-0407.70076

**Published:** 2025-03-26

**Authors:** Asger Ahlmann Bech, Mia Daugaard Madsen, Annika Vestergaard Kvist, Peter Vestergaard, Nicklas Højgaard‐hessellund Rasmussen

**Affiliations:** ^1^ Steno Diabetes Center North Denmark Aalborg University Hospital Gistrup Denmark; ^2^ Department of Clinical Medicine and Endocrinology Aalborg University Hospital Aalborg Denmark

**Keywords:** cardiovascular disease, comorbidity, diabetes complications, major adverse cardiovascular events, multimorbidity, type 2 diabetes mellitus

## Abstract

**Aim:**

This study aimed to investigate the association between cardiovascular risk factors and major adverse cardiovascular events (MACE) in people with type 2 diabetes, while assessing potential changes over time.

**Methods:**

Utilizing data from Danish registries, this study identified people with type 2 diabetes between 2002 and 2021 (*n* = 372 328) and subdivided them into two 10‐year time periods: TP1: 2002–2011 and TP2: 2012–2021, and further categorized into cases and controls. Cases were defined as having suffered a first‐time three‐point MACE (*n*_TP1_ = 12 713, *n*_TP2_ = 8981) and matched 1:1 with controls on age, sex, and type 2 diabetes duration. Exposures were preselected diabetes complications and comorbidities.

**Results:**

Fewer were affected by MACE during TP2 compared to TP1 (*p* < 0.001). Diabetes complications associated with MACE were nephropathy (OR_TP1_ = 1.54, 95% CI 1.30–1.83, OR_TP2_ = 1.47, 95% CI 1.20–1.79), neuropathy (OR_TP1_ = 2.02, 95% CI 1.84–2.21 OR_TP2_ = 1.58, 95% CI 1.44–1.73) and retinopathy (OR_TP1_ = 1.10, 95% CI 0.98–1.23, OR_TP2_ = 1.38, 95% CI 1.17–1.63). Comorbidities associated with MACE included hypertension (OR_TP1_ = 1.30, 95% CI 1.22–1.38 OR_TP2_ = 1.31, 95% CI 1.22–1.41), atrial flutter or fibrillation (OR_TP1_ = 1.46, 95% CI 1.35–1.58, OR_TP2_ = 1.37, 95% CI 1.26–1.50), heart failure (OR_TP1_ = 1.53, 95% CI 1.401.67‐, OR_TP2_ = 1.37, 95% CI 1.23–1.54) and hypercholesterolemia (OR_TP1_ = 1.13, 95% CI 1.07–1.20, OR_TP2_ = 1.02, 95% CI 0.96–1.10). Hypercholesterolemia (*p =* 0.038) and neuropathy (*p =* 0.038) exhibited a significant decrease in association with MACE between the time periods.

**Conclusions:**

The prevalence of first‐time MACE decreased over time, despite a relatively stable prevalence of type 2 diabetes. Several diabetes‐related complications and comorbidities were significantly associated with MACE. The associations of neuropathy and hypercholesterolemia with MACE lessened over time, suggesting potential improvements in risk management or treatment strategies.

## Graphical abstract

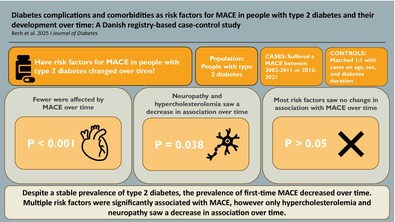

**Summary**

- Prevalence of MACE in people with type 2 diabetes has fallen over time.
- Hypercholesterolemia had a reduced association with MACE over time.

## 1 Introduction

Diabetes mellitus is a metabolic disease characterized by chronic elevated blood glucose [1]. In 2021, an estimated 537 million people were living with diabetes, and the number is expected to increase over the coming years, with the vast majority having type 2 diabetes [2, 3].

Type 2 diabetes increases the risk of developing both macrovascular and microvascular late diabetes complications, with cardiovascular disease (CVD) representing the most severe [3, 4]. Moreover, inadequate glycemic control is linked to various comorbidities, such as hypertension, dyslipidemia, and obesity [3, 5]. These factors contribute to the heightened incidence of CVD in people with type 2 diabetes [3, 5]. Therefore, the prevention and management of these comorbidities have constituted a pivotal emphasis in diabetes care, aiming to diminish the risk of CVD and, consequently, major adverse cardiovascular events (MACE) [6, 7]. MACE has become a progressively utilized outcome of interest in both clinical trials and observational studies, persisting as the primary cause of morbidity and mortality among people affected by CVD [8, 9].

Multimorbidity, defined as the presence of two or more concurrent medical conditions in a person, is prevalent among those with type 2 diabetes [10, 11]. Up to 30% of people with type 2 diabetes exhibit three or more comorbidities at the time of their diabetes diagnosis [11]. It has been speculated that the sum of diabetes complications and the accumulation of different comorbidities together increases the risk of CVD in type 2 diabetes additionally [11, 12, 13, 14]. However, the burden of each comorbidity and the association with CVD, and whether this has changed over time, remain unclear.

Hence, a better understanding of which diseases or conditions affect the risk of developing or potentially reduce the risk of CVD is needed to guide clinicians in treatment regimens to improve the overall quality of life in people with type 2 diabetes. Therefore, it is evident that searching for potential modifiable risk factors is necessary to further decrease the health gap between people with type 2 diabetes and the general population.

Therefore, this study aimed to identify risk factors among people with type 2 diabetes who experienced MACE to ascertain whether these factors have changed over time.

## 2 Methods

### 2.1 Study Design

This study was carried out using a registry‐based case–control design with people over the age of 18 years, diagnosed with type 2 diabetes between 2002 and 2021. People were stratified into two time periods according to the time of diagnosis. Time period 1 (TP1) Included people diagnosed with type 2 diabetes between 2002 and 2011, and time period 2 (TP2) included people diagnosed with type 2 diabetes between 2012 and 2021.

### 2.2 Participants

Type 2 diabetes was defined as diagnosed type 2 diabetes (ICD‐10 code E11) or receiving diabetes medication (ATC code A10A or A10B). Exclusion criteria were type 1 diabetes mellitus (ICD‐10 code E10), other specified diabetes mellitus (ICD‐10 code E13), unspecified diabetes mellitus (ICD‐10 code E14), nondiabetic hypoglycemic coma (ICD‐10 code E15), MACE prior to diagnosis of type 2 diabetes mellitus, and polycystic ovary syndrome (ICD‐10 code E28.2 + ATC code A10BA02, or A10BA02 + GO3C + GO3H, or A10BA02 + G03GB), the latter to reduce the risk of including people without diabetes in the study.

A total of 372,328 people with type 2 diabetes were identified. Of these, 43,988 people were excluded due to a MACE prior to the diagnosis of type 2 diabetes, resulting in 328,340 people with type 2 diabetes included across the two time periods.

**FIGURE 1:**
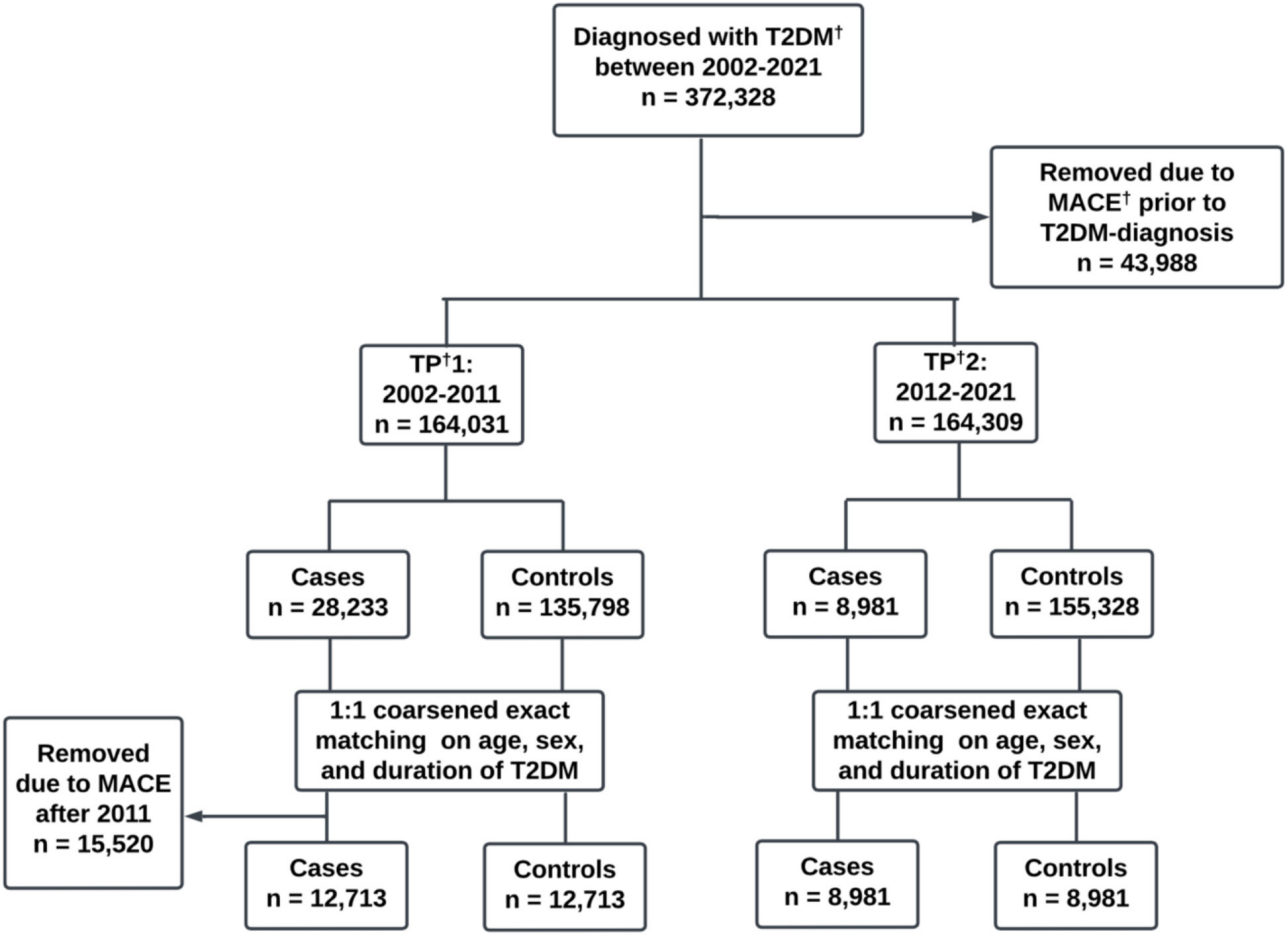
Design flow and number of people included in the study. MACE, major adverse cardiovascular events; T2DM, type 2 diabetes mellitus; TP, time period.

Within each time period, people were further categorized into cases and controls. Cases denoted those who encountered a first‐time MACE during the corresponding 10‐year period, whereas controls encompassed those who did not. In TP1, an additional 15,520 cases were removed due to MACE after 2008, resulting in 12,713 cases for TP1 and 8981 cases for TP2. Cases were matched 1:1 on sex and age with controls, using coarsened exact matching (Figure 1).

### 2.3 Variables

The endpoint was a three‐point MACE, defined as having suffered an acute myocardial infarction (AMI) (ICD‐10: I21), stroke (ICD‐10: I60‐I64), or cardiovascular death (ICD‐10: DI46) [9]. Controls inherited the date of the MACE of their matched case as a dummy date. The duration of type 2 diabetes was defined as the time from diagnosis to the onset of MACE (cases) or dummy date (controls).

Exposures consisted of pre‐selected comorbidities and diabetes complications diagnosed prior to the endpoint, within each of the time periods. The comorbidities were identified using ICD‐10 and/or ATC codes: Hypertension (ICD‐10: I10, ATC: C02, C08, C09), hypercholesterolemia (ICD‐10: E78.0, ATC: C10AA), depression (ICD‐10: F32.0‐DF32.3, F32.8, F32.9, F33.0, F33.4, F33.8, F33.9, ATC: N06AX, N06AA, N06AB), osteoarthritis (ICD‐10: M16, M17), arthritis (ICD‐10: M05.8, M05.9, M06.0, M06.8, M06.9), asthma (ICD‐10: J45.0, J45.1, J45.8, J45.9, J46), atrial fibrillation and atrial flutter (ICD‐10: I48), and heart failure (ICD‐10: I50.0, I50.2, I50.9). The diabetes complications included were retinopathy (ICD‐10: H31.0, H33.4, H35.0‐H35.2, H35.5, H35.7, H36.0, H36.8, E11.3, I70.8), neuropathy (ICD‐10: G56, G57, G58, G59, G60, G61, G62, G63, G90, E11.4, E11.6, M14.6, M49.4), and nephropathy (ICD‐10: E85.0, N08, N07, E11.2, I12.0, I12.9, N14.0, N15.0, N14.3, N14.4, N28.9).

### 2.4 Data Sources

Every person who lives or has lived in Denmark since 1968 has been registered with the Danish Civil Registration System and assigned a 10‐digit CPR number equivalent to a social security number [15]. This unique and individual 10‐digit code is used to identify people in all Danish registries [16]. This enables the identification of all people with type 2 diabetes in Denmark by linking hospital diagnosis data from the Danish National Patient Registry with medication prescription data from the Register of Medicinal Products Statistics of the Danish Medicines Agency.

The Danish National Patient Registry has recorded information on all patients discharged from Danish somatic hospitals since 1977, making it one of the world's oldest nationwide hospital registries [17]. For each patient contact, a primary and optional secondary diagnosis is recorded using the International Classification of Diseases 8th revision (ICD‐8) from 1977 to 1993 and the 10th revision (ICD‐10) from 1994 onwards, as a part of the Health Care Classification System (SKS) [17]. SKS records diagnoses and interventions such as surgery, anesthesia, and examinations [17].

Information of medication was extracted from the Register of Medicinal Products Statistics of the Danish Medicines Agency, which contains individual‐level data on all prescription medications sold in Danish pharmacies since 1996 using the Anatomical Therapeutic Chemical (ATC) classification system [18]. In Denmark, general practitioners handle the diagnostics and treatment of type 2 diabetes, while insufficiently regulated type 2 diabetes and type 1 diabetes are managed by endocrinologists at hospitals.

### 2.5 Statistical Analysis

Descriptive statistics were performed for all variables in each time period. Continuous variables were presented with a mean and standard deviation (SD) or median dependent on data distribution. Categorical data was reported as number (%) of each group. The chi‐square test was used to determine whether the prevalence of comorbidities differed significantly between cases and controls for each time period. The normal distribution of continuous variables was determined using histograms with normal density curves, PP‐ and QQ‐plots. Descriptive statistics were presented for all variables stratified into cases and controls for TP1 and TP2.

Conditional logistic regression was performed for each time period to analyze the association between diabetes complications and comorbidities and the risk of MACE. The models inherently accounted for age, sex, and duration of diabetes through matching. Results were presented as odds ratios (OR) with a 95% confidence interval (CI). A *Z*‐test was used to determine if the odds ratios changed significantly between time periods and to assess the change in prevalence of MACE between time periods. All statistical analyses were performed in Microsoft Excel and STATA version 18 with α set to < 0.05 bilateral.

## 3 Results

### 3.1 Demographics

The mean age was similar in both time periods (TP1: 70.3 years, TP2: 69.4 years), and the proportion of men was higher than that of women (TP1: 62.2% men, TP2: 54.9% men). The duration of type 2 diabetes was lower in TP1 (1.58 years) than in TP2 (2.16 years) (Table 1). The number of people diagnosed with type 2 diabetes was nearly the same between TP1 and TP2, with only a 0.17% increase in TP2.

**TABLE 1:** Person characteristics.

	T1: 2002–2011 (*n* = 25 426)		*p*	T2: 2012–2021 (*n* = 17 962)		*p*
	Cases	Controls		Cases	Controls	
Age (years), mean (SD)	70.27 (12.36)		Matched	69.41 (12.25)		Matched
Sex						
Men (%)	7817 (62.23%)	7817 (62.63%)	Matched	5797 (54.92%)	5797 (54.92%)	Matched
Women (%)	4896 (37.37%)	4896 (37.37%)	Matched	3184 (45.08%)	3184 (45.08%)	Matched
T2D duration (years) (median)	1.58		Matched	2.17		Matched
Diabetic complications						
Nephropathy (%)	400 (3.15%)	228 (1.79%)	< 0.001	282 (3.14%)	164 (1.83%)	< 0.001
Retinopathy (%)	740 (5.82%)	611 (4.81%)	< 0.001	384 (4.28%)	266 (2.96%)	< 0.001
Neuropathy (%)	1634 (12.85%)	840 (6.61%)	< 0.001	1421 (15.82%)	922 (10.27%)	< 0.001
Comorbidities						
Hypertension (%)	9303 (73.18%)	8396 (66.04%)	< 0.001	7030 (78.28%)	6539 (72.81%)	< 0.001
Hypercholesterolemia (%)	6616 (52.04%)	6065 (47.71%)	< 0.001	6193 (68.96%)	6037 (67.22%)	0.013
Atrial flutter or fibrillation (%)	2193 (17.25%)	1429 (11.24%)	< 0.001	1694 (18.86%)	1223 (13.62%)	< 0.001
Heart failure (%)	1731 (13.62%)	1018 (8.01%)	< 0.001	957 (10.66%)	616 (6.86%)	< 0.001
Osteoarthritis (%)	1555 (12.23%)	1567 (12.33%)	0.819	1698 (18.91%)	1662 (18.51%)	0.491
Arthritis (%)	210 (1.65%)	204 (1.60%)	0.766	224 (2.49%)	155 (1.73%)	< 0.001
Asthma (%)	497 (3.91%)	396 (3.11%)	0.001	504 (5.61%)	401 (4.46%)	< 0.001
Depression (%)	3808 (29.95%)	3477 (27.35%)	< 0.001	3317 (36.93%)	3043 (33.88%)	< 0.001
Diabetes treatment						
Biguanides	5908 (46.47%)	6214 (48.88%)	< 0.001	7040 (78.39%)	7252 (80.75%)	< 0.001
Thiazolidinediones	65 (0.51%)	76 (0.60%)	0.353	< 5	< 5	1.00
Sulfonylureas	4817 (37.89%)	4813 (37.86%)	0.959	629 (7.00%)	518 (5.77%)	0.001
DPIVI	241 (1.90%)	258 (2.03%)	0.442	783 (8.72%)	741 (8.25%)	0.261
SGLT2	—	—	—	638 (7.10%)	551 (6.14%)	0.009
GLP1	99 (0.78%)	95 (0.75%)	0.773	438 (4.88%)	453 (5.04%)	0.606
Insulins	982 (7.72%)	891 (7.01%)	0.029	709 (7.89%)	626 (6.97%)	0.018
Other medications						
Statins (%)	6041 (47.52%)	6020 (47.35%)	0.792	5889 (65.57%)	6006 (66.87%)	0.065
ACE/ARB, calcium (%)	8679 (68.27%)	8168 (64.27%)	< 0.001	6700 (74.60%)	6398 (71.81%)	< 0.001
Betablockers (%)	5461 (42.86%)	4474 (35.20%)	< 0.001	4137 (46.06%)	3597 (40.06%)	< 0.001
Diuretics	8152 (64.12%)	7569 (59.54%)	< 0.001	5192 (57.81%)	4815 (53.63%)	< 0.001

*Note:* Study population characteristics for both time periods. Frequency of diabetes complications and comorbidities are given for both cases and controls in each time periods.

Abbreviations: SD, standard deviation; T2DM, type 2 diabetes mellitus; TP, time period.

### 3.2 MACE Prevalence

The prevalence of MACE in TP1 was 8.53%, which decreased significantly to 5.47% in TP2 (*p* < 0.001).

### 3.3 Diabetes Complications

A higher proportion of diabetes complications (i.e., neuropathy, retinopathy, and nephropathy) was observed among cases in comparison to controls in both TP1 and TP2. The prevalence of nephropathy remained consistent across both time periods, while retinopathy slightly decreased from TP1 to TP2. Neuropathy was more prevalent in TP2 (Table 1).

### 3.4 Comorbidities

Hypertension, hypercholesterolemia, atrial fibrillation and atrial flutter, heart failure, asthma, and depression were more frequent in cases than in controls across both time periods. Arthritis was more common among cases in TP2. No discernible disparity was observed for osteoarthritis (Table 1).

In both time periods, hypertension was the most prevalent comorbidity, followed by hypercholesterolemia. Both saw an increase in prevalence from TP1 to TP2 (Table 1).

### 3.5 Medications

Significantly more controls were treated with Biguanides in both TP1 and TP2 compared to cases. Conversely, more cases received insulin treatment in both time periods. The use of other diabetes medications showed no significant difference between cases and controls in both time periods (Table 1).

There was no significant difference in the use of statins between cases and controls in either time period. In contrast, antihypertensive medications (ATC: C02, C08, C09), beta‐blockers, and diuretics were used more frequently in cases than in controls across both time periods (Table 1).

### 3.6 Conditional Logistic Regression

Neuropathy, nephropathy, retinopathy, hypertension, hypercholesterolemia, atrial fibrillation or atrial flutter, and heart failure were associated with an increased risk of MACE. Osteoarthritis was significantly inversely associated with MACE in TP1. No significant association with MACE was found for retinopathy and arthritis in TP1, and hypercholesterolemia and osteoarthritis in TP2 (Table 2).

**TABLE 2:** Conditional logistic regression for each time period with MACE^a^ as outcome.

	TP^a^1: 2002–2011		TP^a^2: 2012–2021		*Z‐score*	*p*
	OR^a^ (95% CI^a^)	*p*	OR^a^ (95% CI^a^)	*p*		
T2D complications						
Nephropathy	1.54 (1.30–1.83)*	< 0.001	1.47 (1.20–1.79)*	< 0.001	0.24	0.810
Retinopathy	1.10 (00.98–1.23)	0.121	1.38 (1.17–1.63)*	< 0.001	−1.75	0.079
Neuropathy	2.02 (1.84–2.21)*	< 0.001	1.58 (1.44–1.73)*	< 0.001	2.07	0.038*
Comorbidities						
Hypertension	1.30 (1.22–1.38)*	< 0.001	1.31 (1.22–1.41)*	< 0.001	−0.19	0.848
Hypercholesterolemia	1.13 (1.07–1.20)*	< 0.001	1.02 (0.96–1.10)	0.516	2.08	0.038*
Atrial flutter or fibrillation	1.46 (1.35–1.58)*	< 0.001	1.37 (1.26–1.50)*	< 0.001	0.73	0.464
Heart failure	1.53 (1.40–1.67)*	< 0.001	1.37 (1.23–1.54)*	< 0.001	1.03	0.303
Osteoarthritis	0.91 (0.84–0.98)*	0.018	0.94 (0.87–1.02)	0.132	−0.66	0.509
Arthritis	0.87 (0.71–1.07)	0.193	1.37 (1.11–1.70)*	0.004	−2.57	0.010*
Asthma	1.16 (1.01–1.34)*	0.037	1.17 (1.02–1.34)*	0.025	−0.07	0.948
Depression	1.09 (1.03–1.15)*	0.004	1.10 (1.03–1.17)*	0.003	−0.27	0.788

*Note:* Results of conditional logistic regression for each time period. Change in association with MACE between time periods was determined using *Z*‐test. The models inherently accounted for age, sex, and type 2 diabetes duration through matching.

^a^ Abbreviations: CI, confidence interval; MACE, major adverse cardiovascular events; OR, odds ratio; T2DM, type 2 diabetes mellitus; TP, time period.

* Statistically significant result.

Neuropathy (*p* = 0.038) and hypercholesterolemia (*p* = 0.038) were the sole conditions displaying a significantly reduced association between time periods. Conversely, arthritis showed a significant increase in association with MACE in TP2 (*p* = 0.010).

## 4 Discussion

In this case–control study, using data from the Danish National Patient Registry, findings revealed a significant decrease in the overall prevalence of MACE among people with type 2 diabetes between the two time periods. Diabetic complications and comorbidities like hypertension, heart failure, and atrial flutter or fibrillation were most associated with MACE in both time periods, based on odds ratios. The association of neuropathy and hypercholesterolemia with MACE lowered between time periods. Additionally, osteoarthritis exhibited an inverse association with MACE in the first time period.

### 4.1 Diabetes Duration and Treatment Patterns

In TP2, people had been diagnosed with type 2 diabetes longer than in TP1 at the occurrence of MACE. A plausible explanation could be improvements in the early detection of type 2 diabetes and a general reduction in cardiovascular mortality, as well as the implementation of HbA1c as a diagnostic tool in 2012 [19, 20]. The use of anti‐diabetic drugs also differed significantly, with more cases being treated with insulin and more controls being treated with biguanides in both time periods. This suggests that the cases had more advanced type 2 diabetes compared to the controls, potentially contributing to their increased risk of MACE in some of the cases.

### 4.2 Diabetes Complications

The prevalence of nephropathy, retinopathy, and neuropathy was significantly higher in cases than in controls in both time periods. The prevalence of neuropathy in this study (6%–15%) was higher than the anticipated rates for Danish individuals with type 2 diabetes (≈1.2%), other studies report higher prevalence rates [21, 22, 23]. Conversely, the prevalence of retinopathy was lower in our study (2.96%–5.8%) compared to the expected (≈13%) [20]. Lastly, the prevalence of nephropathy (1.8%–3.15%) was also lower than the expected (≈5%–10%) [24].

Nephropathy, retinopathy, and neuropathy represented risk factors with increased association with MACE. This is in line with Yap et al., a prospective cohort study that investigated diabetes, glycemic control, and microvascular complications (retinopathy and nephropathy) as predictors for MACE [25]. The study found systolic blood pressure, nephropathy, and retinopathy as significant predictors for MACE. However, retinopathy became insignificant after adjusting for confounders [25]. A cohort study by Brownrigg et al. found peripheral neuropathy was significantly associated with incidents of CVD after adjusting for confounders, in people with type 2 diabetes [26]. Other studies investigating the relationship between diabetes microvascular complications and cardiovascular outcomes showed similar results [27, 28, 29, 30]. From TP1 to TP2, a decline in prevalence was seen in retinopathy, while neuropathy increased, and nephropathy remained largely unchanged. Generally, neuropathy is an underdiagnosed complication, and the increase seen could be due to increased focus on the diagnosis of neuropathy [30]. Meanwhile the decline in retinopathy and the stability in nephropathy might be explained by improved preventive measures. Our findings further supported that people with type 2 diabetes and microvascular complications have an increased risk of macrovascular complications. However, only neuropathy showed a significant decrease in odds ratio between the time periods, indicating that improved management is still needed. However, when interpreting these results, it is important to consider the general unreliability of neuropathy diagnosis and reporting.4.3 Comorbidities.

A significantly higher proportion of hypertension, atrial flutter or fibrillation, and heart failure were present in cases than in controls, with the prevalence increasing from TP1 to TP2. Hypertension, atrial flutter or fibrillation, and heart failure were also the comorbidities with the highest odds ratios for MACE in TP1. However, when comparing the two time periods, no significant change in association was seen. The only comorbidity with a significant decrease in odds ratio from TP1 to TP2 was hypercholesterolemia. The reduced odds ratio for hypercholesterolemia could be due to improvements in treatment and guidelines, with many important studies taking place in the 2000s and new guidelines for the treatment of dyslipidemia being released in the early 2010s for both Europe and North America [31, 32, 33]. A review by the U.S. Preventive Services Task Force (USPSTF) concluded that statin therapy as a primary prevention strategy for cardiovascular disease (CVD) effectively reduced both mortality and adverse cardiovascular outcomes in adults with increased risk of CVD without a prior history of CVD [34]. This is supported by multiple studies demonstrating that statin therapy reduces the risk of cardiovascular events in individuals without prior CVD [35, 36, 37]. Also, in the mid to late 2000s, a vast number of studies were conducted to improve the treatment of hypertension in different populations [38]. Guidelines for hypertension management were updated in both 2007 and 2013 [39]. One would expect this to have benefited people in TP2 more than in TP1. However, our results do not show any change in association with MACE. While new generations of antihypertensive and cholesterol‐lowering drugs did emerge, the majority of changes in guidelines were concerning when to initiate treatment, rather than what to treat with [31, 39]. For hypercholesterolemia, cases saw an 18% increase in the use of statins and controls a 19% increase from TP1 to TP2. This increase could explain the significantly reduced odds ratio. In contrast, the odds ratio of MACE in people with hypertension remained unchanged, suggesting that there is still room for further improvement.

Atrial fibrillation and atrial flutter, as well as heart failure, were also more common in cases than in controls in both time periods and associated with an increased odds ratio for MACE, which is similar to other study findings [40, 41]. In a prospective cohort by Soliman et al. atrial fibrillation was found to significantly increase the risk of AMI after adjusting for confounders [40]. Heart failure is also associated with an increased risk of death due to arrhythmias (i.e., leading to sudden cardiac death) and CVD [41]. Similarly to diabetes complications, no significant change in odds ratios was observed between TP1 and TP2 for atrial fibrillation, atrial flutter, or heart failure.

Our study also found that depression increased the odds ratio of MACE. This is in line with a systematic review and meta‐analysis by Farooqi et al. examining depression and cardiovascular risk in people with type 2 diabetes, which found that people with type 2 diabetes and depression had an increased risk of both coronary heart disease and stroke, compared to people with type 2 diabetes without depression [42]. The reason for this is not fully understood; however, it is believed that both antidepressant treatment and lifestyle linked with depression (i.e., eating unhealthier, exercising less, etc.) account for this increased risk [43].

Furthermore, asthma in both time periods and arthritis in TP2 were found to be significantly associated with MACE. Other studies have linked both asthma and arthritis to increased risk of CVD, potentially through systemic inflammation or medications used in the treatment of the diseases [44, 45, 46, 47].

Osteoarthritis had an inverse association with MACE in TP1, which is in contrast to other studies, where osteoarthritis has previously been identified as a significant risk factor for CVD [48, 49]. A possible explanation for the inverse association could be that in more severe cases of CVD, this diagnosis may have been prioritized over osteoarthritis, as proposed by another study on osteoarthritis and CVD [50].

### 4.3 Strengths

This study had several strengths when comparing the association between different diabetes complications and comorbidities, and the risk of suffering MACE compared to controls over time (i.e., TP1 vs. TP2). First, the case–control design is a useful study type when examining multiple exposures' influence on a certain outcome and can provide evidence of associations to further investigate [51]. Second, multiple potential confounders were accounted for, as both cases and controls were matched on age, sex, and duration of type 2 diabetes. Third, as our population was selected based on the same inclusion and exclusion criteria for both TP1 and TP2, this study enabled comparison of the two time periods as the groups were compatible. Lastly, the use of the Danish National Patient Registry was a strength as the registry, even though relying on manual registration by clinicians, is known for high validity [17]. The use of registries also eliminated the risk of recall bias, as no recalling of exposures from participants was used.

### 4.4 Limitations

This study found multiple comorbidities having a significant association with MACE in type 2 diabetes. However, some limitations must be considered. The lack of additional variables, such as HbA1c, smoking status, body mass index, and socioeconomic factors, was a major limitation, as these could all be confounders. A second limitation was the adoption of HbA1c as a diagnostic criterion in 2012, which may have introduced variability across the time periods [20]. However, the large sample size likely mitigated the impact of this change in diagnostics. Additionally, the size of the study population in both time periods was similar. A third limitation was the use of 10‐year time periods, as it reduces the study population, as seen by the removal of over half of all cases with MACE in TP1, due to suffering MACE outside of their time period. The inclusion of these people could potentially have given valuable information about the association between the diabetes complications, comorbidities, and MACE. This would, however, reduce the comparability of the two time periods, as TP1 would have had twice as long to develop MACE and TP1 would potentially benefit from the same developments in guidelines and treatments as TP2.

## 5 Conclusions

This study found that in people with type 2 diabetes, the prevalence of MACE had decreased from TP1 to TP2. Furthermore, multiple risk factors were found to be associated with MACE. Diabetic neuropathy and hypercholesterolemia were significantly associated with increased odds of MACE, but the odds ratios decreased from TP1 to TP2. Conversely, diabetic nephropathy, hypertension, atrial fibrillation and atrial flutter, and heart failure were also associated with increased odds of MACE, but odds ratios did not change significantly between the time periods.

Overall, these findings highlight several comorbidities as potential modifiable risk factors in people with type 2 diabetes, which can be targeted to reduce the risk of MACE.

## Author Contributions

**Asger Ahlmann Bech:** conceptualization, methodology, formal analysis, investigation, writing – original draft preparation, visualization, project administration. **Mia Daugaard Madsen:** conceptualization, methodology, formal analysis, investigation, writing – original draft preparation, visualization, project administration. **Annika Vestergaard Kvist:** methodology, formal analysis, investigation, data curation, writing – review, and editing. **Peter Vestergaard:** conceptualization, methodology, writing – review, and editing, supervision, project administration. **Nicklas Højgaard‐hessellund Rasmussen:** conceptualization, methodology, formal analysis, investigation, writing – review, and editing, supervision, project administration.

## Ethics Statement

This study is based on epidemiological data and does not involve human participants or identifiable personal data. Therefore, ethical approval was not required.

## Conflicts of Interest

Mia D. Madsen holds shares in Novo Nordisk. Peter Vestergaard is head of research at the Steno Diabetes Center North Denmark, sponsored by the Novo Nordisk Foundation. Nicklas H. Rasmussen holds shares in Novo Nordisk, a grant from DDEA, and lecture fees from Boehringer Ingelheim. Asger A. Bech and Annika V. Kvist declare no conflicts of interest.

## Data Availability

The data sets used in the study are not available for sharing because of Danish national regulations. The code used for the study analysis can be made available upon reasonable request.
